# An *in silico* platform for the design of heterologous pathways in nonnative metabolite production

**DOI:** 10.1186/1471-2105-13-93

**Published:** 2012-05-11

**Authors:** Sunisa Chatsurachai, Chikara Furusawa, Hiroshi Shimizu

**Affiliations:** 1 Department of Biotechnology, Graduate School of Engineering, Osaka University, 2-1 Yamadaoka, Suita, Osaka, 565-0871, Japan; 2 Quantitative Biology Center, RIKEN, 6-2-3 Furuedai, Suita, Osaka, 565-0874, Japan; 3 Department of Bioinformatics Engineering, Graduate School of Information Science and Technology, Osaka University, 1-5 Yamadaoka, Suita, Osaka, 565-0871, Japan

## Abstract

**Background:**

Microorganisms are used as cell factories to produce valuable compounds in pharmaceuticals, biofuels, and other industrial processes. Incorporating heterologous metabolic pathways into well-characterized hosts is a major strategy for obtaining these target metabolites and improving productivity. However, selecting appropriate heterologous metabolic pathways for a host microorganism remains difficult owing to the complexity of metabolic networks. Hence, metabolic network design could benefit greatly from the availability of an *in silico* platform for heterologous pathway searching.

**Results:**

We developed an algorithm for finding feasible heterologous pathways by which nonnative target metabolites are produced by host microorganisms, using *Escherichia coli*, *Corynebacterium glutamicum*, and *Saccharomyces cerevisiae* as templates. Using this algorithm, we screened heterologous pathways for the production of all possible nonnative target metabolites contained within databases. We then assessed the feasibility of the target productions using flux balance analysis, by which we could identify target metabolites associated with maximum cellular growth rate.

**Conclusions:**

This *in silico* platform, designed for targeted searching of heterologous metabolic reactions, provides essential information for cell factory improvement.

## Background

Recognizing the potential depletion of petroleum resources, researchers have become increasingly interested in production of fuels and industrial chemicals by microorganisms [1-3]. Such biosnythesized materials include fuels, plastics, polymers, food additives, feed additives, solvents and drugs [4-6]. For example, ethanol and higher alcohols are used as fuels and solvents in a wide variety of chemical processes [7]. 1,3-propanediol forms the basis of polymers such as polytrimethylene terephthalate (PTT) [8], while isoprene is an intermediate metabolite in the production of cis-1,4-polyisoprene, a synthetic of natural rubber [9]. To produce such industrially useful materials, modifications of host metabolic systems are generally required. Target metabolites are frequently produced by incorporating heterologous metabolic pathways into well-characterized host microorganisms, such as *Escherichia coli, Saccharomyces cerevisiae*, and *Corynebacterium glutamicum *[10-15]. However, the selection of suitable heterologous metabolic pathways for host organisms is hindered by metabolic network complexity. Although copious data on metabolic reactions have been amassed in the literature and in public databases, such as KEGG [16], BRENDA [17], and ENZYME [18], constructing a target production pathway from a host metabolic network while maintaining the required metabolic balances in the host (e.g., nicotinamide adenine dinucleotide (NADH) production/consumption) requires a researcher’s experience and intuition. Thus, the development of an appropriate *in silico* platform will enhance industry-focused metabolic network design by providing possible heterologous pathways for target metabolite production.

In recent years, several *in silico* heterologous pathway search methods have been proposed and used in target metabolite production [19-30]. Some of these predict metabolic pathways based on chemical transformation patterns between the substrate and the product [19,20,24,25]. For example, PathMiner [19] heuristically determines metabolic pathways from known enzyme-catalyzed transformations, by minimizing pathway costs. PathPred [29] extracts biochemical structural transformation patterns from databases, from which plausible pathways can be constructed even if no reactions that directly generate the target metabolites are known. By supplying information about reactions, PathPred enables the user to create a metabolite that is structurally similar to the target.

Several graph-based methods for heterologous pathway search are also available [21-23,26,28,30]. OptStrain [30] utilizes mixed integer linear programming to identify heterologous reactions, producing a target that satisfies the stoichiometric balance while minimizing the number of heterologous reactions. Following stoichiometric addition of the heterologous reactions, the OptKnock [31] algorithm maximizes the target productivity. As another example, novel metabolic routes have been efficiently screened by probabilistic selection of metabolic pathways [27]. Although several methods exist for screening heterologous pathways of target metabolite production, there remains a lack of consensus on how to choose heterologous pathways and host microorganisms for target production. Heterologous reaction screening generally requires extensive calculations; thus, it is difficult to compare the screening results. In this study, to avoid such calculations, we developed a simple *in silico* screening platform to identify feasible heterologous pathways of nonnative target metabolite production.

We first developed a pathway search algorithm that identifies the shortest pathway between a host metabolic network and target metabolites as heterologous reactions are added. Using this algorithm, we screened all producible target metabolites listed in databases by adding heterologous reactions to host microorganisms. For all producible target metabolites, we then estimated the production yields using flux balance analysis (FBA), assuming steady-state conditions and maximum biomass production rate. By analyzing the entire list of producible target metabolites in several different hosts, we selected a set of rational heterologous pathways and host microorganisms that will likely produce desired targets.

## Methods

### Construction of an in-house database of metabolic reactions

All known metabolic reactions were considered as candidate heterologous reactions that could be added to the host metabolic network. We first constructed an in-house database of metabolic reactions from data stored in the KEGG ligand section [16] and BRENDA [17] databases. All metabolic reaction information regarding genes, enzymes, pathways, and organisms in the KEGG database was collected into the database, which was developed using PostgreSQL 9.0 (The PostgreSQL Global Development Group). The Michaelis-Menten constants (*K*_*m*_) of the enzymatic reaction data were retrieved from BRENDA [17]. We also used Python scripts to access the in-house database.

### Genome-scale metabolic model of host microorganisms

In this study, we adopted 3 host microorganisms widely used in industry; namely, *E. coliC. glutamicum*, and *S. cerevisiae*. *E. coli* has been exploited for such industrially valuable compounds as L-phenylalanine, L-tyrosine, 1-butanol and 1,2-propanediol [32-34]. *C. glutamicum* is widely used in amino acid production [35]. *S. cerevisiae* is an important producer of alcohols and organic acids such as lactate [36]. These organisms are ideal hosts of bioengineered products since they exhibit high growth activity under various conditions and are easily genetically manipulated [37,38].

We used genome-scale metabolic models of *S. cerevisiae* (iMM904) [39], *E. coli* (iJR904) [40], and *C. glutamicum*[41], based on earlier metabolic constructions [39][41]with slight modifications. Because our pathway search algorithm uses the heterologous reactions listed in the KEGG database, all metabolite IDs in the earlier genome-scale metabolic models were converted to the KEGG compound ID format using metabolite name matching by manual checking.

### Heterologous pathway identification for target production

We developed an algorithm to identify heterologous reaction(s) producing a target metabolite within a host microorganism. The algorithm expands the host metabolic network by sequentially adding heterologous metabolic reactions from our in-house database. The heterologous pathway identification procedure is as follows:

1. A set of metabolites M0 and a set of metabolic reactions R0 are defined as those present in the genome-scale metabolic network of the host microorganism.

2. From the in-house database, heterologous reactions that satisfy the following conditions are collected: (i) the reaction does not exist in R0, and (ii) it can produce metabolites that do not exist in M0 from a metabolite in M0. A set of these heterologous reactions is defined as R1, and a set of metabolites produced by reactions in R1 is defined as M1.

3. In the same way, Ri is the set of reactions not present in {R0, R1, … , Ri − 1} which can produce metabolites not existing in {M0, M1, … , Mi − 1} from metabolites included in those sets. This expansion procedure is iterated until no further reaction is connectable to the expanded metabolic network.

If a target metabolite is included in a nonnative metabolite set *M*_*i*_, we can identify a set of heterologous reactions that are necessary to produce the target metabolite. For simplicity, all metabolic reactions in the database were assumed to be reversible. Of course some reactions are known to be irreversible, such as the carboxylation and decarboxylation reactions classified by Nomenclature Committee of the international Union of Biochemistry and Molecular Biology (NC-IUBMB) [42]. However, for the majority of reactions in the database, directional information is limited and thus the reversibility of the reactions is difficult to judge. By assuming that all reactions are reversible, we avoid the risk of missing important heterologous pathways due to misjudgment of their reaction reversibility. Our strategy here is to initially screen all possible heterologous pathways regardless of reaction irreversibility, then decide whether the predicted pathway is plausible based on physiological knowledge of the reaction irreversibility.

### Flux balance analysis

FBA is based on a genome-scale metabolic model and optimization of a specific objective flux by linear programming [43,44]. We used FBA to estimate the metabolic flux profile of metabolic networks expanded with heterologous reactions. A pseudo-steady state is assumed, that is, the net sum of all production and consumption fluxes for each internal metabolite is zero. In matrix notation, this condition is represented as S⋅v=0_,_ where  is the stoichiometric matrix representing the stoichiometry of metabolic reactions in the network and  is the vector of metabolic fluxes. In FBA, the flux profile (constrained by steady state) is determined by optimizing a specific objective function. The biomass production flux is one of several widely used objective functions that can be maximized. The flux profiles obtained by maximizing biomass production fluxes are known to be well correlated with those obtained experimentally [39-41,45].

In this study, the coefficients of metabolites representing biomass production flux were extracted from earlier studies [39-41]. We employed another objective function, the production flux of the target metabolite, to judge whether the target metabolite was producible by the metabolic network. In all of the FBA simulations in this paper, glucose was chosen as the sole carbon source and the following external metabolites were allowed to freely transport through the cell membrane: CO_2_, H_2_O, SO_4_ or SO_3_, and NH_3_. All calculations were performed using MATLAB 2009b (MathWorks Inc., Natick, MA). The linear programming problem was solved using GLPK 4.34 (GNU Linear Programming Kit) [46] via the MATLAB interface.

## Results and discussion

### Identification of heterologous pathway(s)

**Figure 1:**
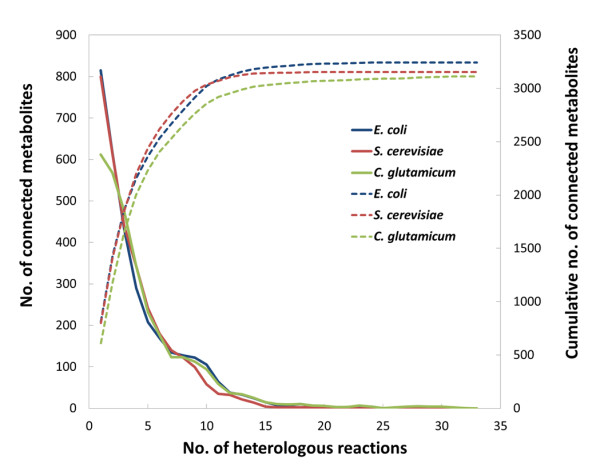
**Number of connected nonnative metabolites produced by heterologous reactions in 3 host microorganisms.** The first vertical axis (solid line) shows the number of connected metabolites in each iteration, while the second vertical axis (dotted line) shows the cumulative number of the connected metabolites.

7,769 metabolic reactions and 6,635 metabolites (shown in the Additional file 1) from 1,139 species were collected from the KEGG database and deposited in our in-house database. To screen for target metabolites that could be produced by our host microorganisms *S. cerevisiae, E. coli,* and *C. glutamicum*, we iteratively expanded the host metabolic network by adding heterologous metabolic reactions as described in the Methods section. Figure 1 displays the number of nonnative metabolites connected to the host metabolic network as a function of the number of heterologous reactions. Fewer than 33 heterologous reactions are required to connect 3,154, 3,244, and 3,112 nonnative metabolites to the host metabolic networks of *S. cerevisiae, E. coli,* and *C. glutamicum* respectively.

The list of metabolites connected to the host metabolic networks is presented in the 2,3,4. To this list, we added the *K*_m_ values of heterologous enzymes. Knowing the *K*_m_ assists in deciding which heterologous enzymes originating from various organisms should be introduced to the host. The names of organisms in the BRENDA database displaying minimum *K*_m_ of the corresponding heterologous enzymes are also listed [17], since the enzyme from this organism is expected to have highest affinity among the orthologous enzymes to the corresponding substrate. Importantly, these identified heterologous reactions of nonnative metabolite production agreed well with those widely used in metabolic engineering and which are important to the industry (Table 1), such as isoprene, α-farnesene, poly-β-hydroxybutyrate (PHB), and cadaverine.

**Table 1:** Examples of nonnative metabolites for which our algorithm detected heterologous reactions matching those of previous studies

**Compound (synonym separated by a semicolon)**	**KEGG ID**	**Heterologous reaction(s) from the literature**	**Reference**	**Evaluation of *in silico* design**
**Isoprene; 2-methyl-1,3-butadiene**	C16521	Introduced ispS gene from *Populus nigra* to *Escherichia coli*	[47]	Identical reaction found in *E. coli* and in *Saccharomyces cerevisiae* and *Cerevisiae glutamicum* as the host
**α-Farnesene**	C09665	Introduced farnesene synthase from plant to *E. coli*	[13]	Identical reaction found in *E. coli* and in *S. cerevisiae* and *C. glutamicum* as the host
**Poly-β-hydroxybutyrate; PHB**	C06143	Introduced phbC and phbB from *Streptomyces aureofaciens* to *E. coli*	[48]	Identical reaction found in *E. coli* and in *S. cerevisiae* and *C. glutamicum* as the host
**Cadaverine; 1,5-pentanediamine; 1,5-diaminopentane**	C01672	Introduced ldcC from *E. coli* to *C. glutamicum*	[35,49]	Identical reaction found in *C. glutamicum* and in *S. cerevisiae* as the host
**Amorpha-4,11-diene**	C16028	Introduced AMS1 from the plant *Artemisia annua L.* to *E. coli*	[50,51]	Identical reaction found in *E. coli* and *S. cerevisiae* and in *C. glutamicum* as the host
**Propane-1,3-diol; 1,3propanediol; trimethylene glycol**	C02457	Introduced glycerol dehydratase and 1,3-propanediol oxidoreductase from *Klebsiella pneumonia* to *E. coli*.	[52,53]	Identical reaction found in *E. coli* and in *S. cerevisiae* as the host
**Ethanol; ethyl alcohol; methylcarbinol**	C00469	Introduced pyruvate decarboxylase and alcohol dehydrogenase genes from *Zymomonas mobilis* to *C. glutamicum*	[54]	Identical reaction found in *C. glutamicum* as the host
**(R,R)-Butane-2,3-diol; (R,R)-2,3-Butanediol; (R,R)-2,3-Butylene glycol**	C03044	Introduced acetolactate decarboxylase and butanediol dehydrogenase genes to *E. coli*	[55]	Identical reaction found in *E. coli* as the host
**(R)-Propane-1,2-diol; (R)-1,2-propanediol; (R)-propylene glycol**	C02912	Introduced glycerol dehydrogenase gene from *Klebsiella pneumonia* and used aldehyde dehydrogenase to produce product in *E. coli*	[56]	Alternative pathway found to produce target by adding methylglyoxal reductase and lactaldehyde reductase to *E. coli*
		Introduced glycerol dehydrogenase and methylglyoxal synthase genes from *E. coli* to *S. cerevisiae*	[57]	Alternative pathway found to produce target by adding methylglyoxal reductase and lactaldehyde reductase to *S. cerevisiae*
**Itaconate; itaconic acid; methylenesuccinic acid**	C00490	No information	NA	EC 4.2.1.4-citrate dehydratase and EC 4.1.1.6-aconitate decarboxylase were found to be added to *E. coli* as the host.
** *cis* , *cis* -Muconate; *cis* , *cis* -hexadienedioate; *cis* , *cis* -2,4-hexadienedioic acid**	C02480	Introduced aroZ, aroY, and catA to *E. coli*	[58]	Alternative pathways from antharnilate or 2,3-dihydroxybenzoate to produce catechol, which is a substrate for *cis*,*cis*-muconate production
**Adipate; adipic acid; hexanedioate; hexan-1,6-dicarboxylate**	C06104	Introduced aroZ, aroY, and catA to *E. coli* for producing *cis*,*cis*- muconate and then convert to adipic acid by chemical synthesis	[58]	Alternative pathway found to produce the target by adding 5 heterologous reactions to *E. coli* or *C. glutamicum* as the hosts (see Additional files 5 and 6 for enzyme information)

**Figure 2:**
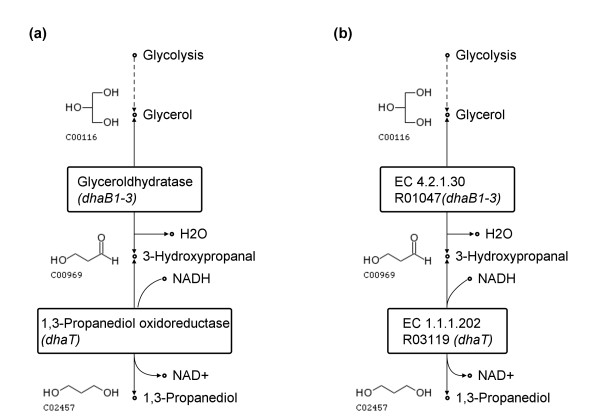
**Heterologous pathways for 1,3-propanediol production: (a) the production pathway described in earlier studies, in *Escherichia coli* [**[52,53]**]; (b) the pathway identified by our algorithm in either *E. coli* or *Saccharomyces cerevisiae* as the host.**

As an example, the production pathways of 1,3-propanediol (C02457) by *E. coli* and *S. cerevisiae*, which were adopted in earlier studies [52,53], are shown in Figure 2. In the previous studies, C02457 production proceeded via conversion of glycerol to 3-hydroxypropanal using glycerol dehydratase (encoded by *dhaB1-B3*). 1,3-Propanediol was then produced, aided by 1,3-propanediol oxidoreductase (encoded by *dhaT*). In this study, the screened heterologous pathways for C02457 production exactly matched those of the earlier studies. In *E. coli*, the screened production pathways of isoprene, α-farnesene, and PHB derived by our algorithm were also identical to those of the earlier studies, while similar heterologous genes introduced to the alternative hosts (*C. glutamicum* and *S. cerevisiae*) additionally produced these targets (see Table 1). Moreover, both reported and alternative production pathways were screened by our algorithm. For instance, we found that *E. coli* cells can produce (R)-propane-1,2-diol when methylglyoxal reductase and lactaldehyde reductase are added to the metabolic network, which has not been reported to date. Similar alternative pathways were found for the production of itaconate, *ciscis*-muconate, and 2,3-dihydroxybenzoate. These results suggest that our algorithm successfully identified the metabolic reactions necessary for the target productions and could assist in screening for potential host cells.

Next, we used glucose as a carbon source to investigate whether these nonnative metabolites are producible by FBA simulations. In this simulation, the production flux of each nonnative metabolite was treated as an objective function to be maximized under the steady-state assumption. When the maximum production flux of a nonnative metabolite is zero, this metabolite is non-producible under the given condition.

We calculated the maximum production fluxes of all connectable nonnative metabolites. 28% of the connectable nonnative metabolites of *E. coli* could not be produced using glucose as a sole carbon source. Similarly, 33% of the connectable nonnative metabolites of *S. cerevisiae* and 16% of the connectable nonnative metabolites of *C. glutamicum* were non-producible under this condition. These non-producible metabolites were identified by their tendency to disconnect when glycolysis formed the central metabolic pathway. In *E. coli*, these metabolites included *trans*-aconitate (C02341), butyrate (C00246), acetoacetate (C00164), and l-lactaldehyde (C00424).

### Evaluation of production feasibility

To evaluate the feasibility of nonnative target metabolite production, we performed FBA simulations under conditions of maximizing biomass production following heterologous reaction expansion of the genome-scale metabolic model. Metabolic flux profiles calculated at maximum biomass production rates have been shown to closely represent those in real microorganisms [45,59-62]. Such agreement may be explained by the growth optimization of microorganisms through evolutionary dynamics [63]. Furthermore, for the mutant strains constructed in the laboratory, the cells could achieve the near-optimal metabolic state calculated by the FBA simulation after long-term cultivation [64-67], via the selection of faster growing cells. Thus, we can expect that if a nonnative target metabolite is produced in the FBA simulation under maximized biomass production, that target may be feasibly manufactured.

**Figure 3:**
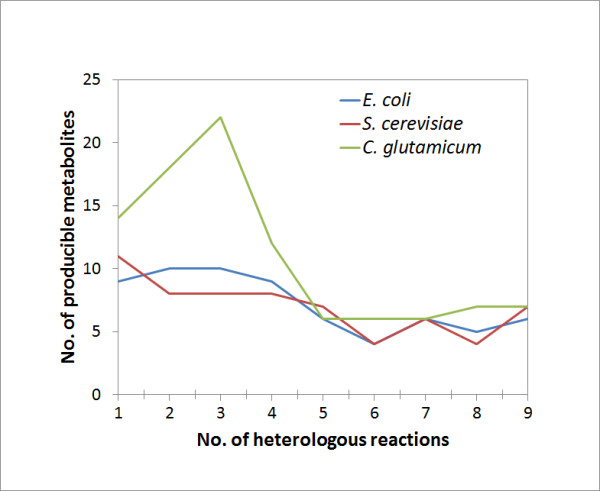
The number of metabolites producible under biomass maximization conditions with the addition of <10 heterologous reactions.

In Figure 3, we plot the number of target metabolites produced under maximized biomass production, versus the number of heterologous reactions necessary for metabolite production. We set a threshold yield (1%) to identify the produced metabolites because the production yields of some metabolites were positive but extremely small. Sometimes the FBA solution was undetermined under biomass maximization conditions; that is, the solution was not unique. In such cases, following maximization of biomass production, the production flux of the target metabolites was further maximized with fixing the maximized biomass production, to obtain a unique flux profile that would generate the target. In the simulations, we adopted a micro-aerobic condition to screen the target metabolites produced under the biomass maximization condition, in which significantly more metabolites were obtained than under anaerobic conditions, and in which all anaerobically produced metabolites were included.

Table 2 lists the representative target metabolites produced under biomass maximization, together with their corresponding heterologous reactions. The mechanisms involved in these reactions can be classified into two categories. One is based on the production of oxygen as a by-product of the targets. Since the simulations were performed under micro-aerobic conditions, oxygen supply increased the biomass production by activating the electron transfer system and facilitating adenosine triphosphate production. Therefore, if the heterologous reactions used to produce the target are accompanied by oxygen production, the target can be produced under minimum biomass production flux. For example, pentane-2,4-dione was produced by introducing a single heterologous reaction into *E. coli* and *S. cerevisiae*, whereas two heterologous reactions were necessary to produce this metabolite in *C. glutamicum*. Vanillin can be produced under the same mechanism by introducing 4 heterologous reactions into the *E. coli* and *C. glutamicum* metabolic networks.

**Table 2:** Examples of producible nonnative metabolites under conditions of maximized biomass production

**Nonnative metabolites**	**Host network**	**By-product**	**No. of reaction(s)**	** Heterologous reaction(s)**	**EC number**
**Pentane-2,4-dione**	*E. coli, S. cerevisiae*	Oxygen	1	Pentane-2,4-dione + oxygen ↔ acetate + methylglyoxal	1.13.11.50
	*C. glutamicum*	Oxygen	2	Glycerone phosphate ↔ methylglyoxal + orthophosphate	4.2.3.3
				Pentane-2,4-dione + oxygen ↔ acetate + methylglyoxal	1.13.11.50
**Vanillin (4-hydroxy-3-methoxy -benzaldehyde)**	*E. coli, C. glutamicum*	Oxygen, NADH	4	Formaldehyde + NAD+ + H_2_O ↔ formate + NADH + H^+^	1.2.1.46
				3-Dehydroshikimate ↔ 3,4-dihydroxybenzoate + H2O	4.2.1.118
				Vanillate + oxygen + NADH + H^+^ ↔ 3,4-dihydroxybenzoate + NAD + + H_2_O + formaldehyde	1.14.13.82
				Vanillate + NAD+ + H2O ↔ 4-hydroxy-3-methoxy-benzaldehyde + oxygen + NADH + H^+^	1.2.3.9
**(R)-Propane-1,2-diol**	*E. coli*	NAD^+^	2	(R)-Lactaldehyde + NAD^+^ + H_2_O ↔ (R)-lactate + NADH + H^+^	1.2.1.23
				(R)-Propane-1,2-diol + NAD^+^ ↔ (R)-lactaldehyde + NADH + H^+^	1.1.1.77
**2-Propyn-1-al**	*S. cerevisiae*	NAD^+^	3	3-Oxopropanoate ↔ acetaldehyde + CO_2_	4.1.1.-
				3-Oxopropanoate ↔ propynoate + H_2_O	4.2.1.27
				2-Propyn-1-al + NAD^+^ + H_2_O ↔ propynoate + NADH + H^+^	1.2.1.3
**Adipate semialdehyde**	*E. coli*	NADP+	6	Succinyl-CoA + acetyl-CoA ↔ CoA + 3-oxoadipyl-CoA	2.3.1.174
				(3 S)-3-Hydroxyadipyl-CoA + NAD^+^ ↔ 3-Oxoadipyl-CoA + NADH + H^+^	1.1.1.35
				5-Carboxy-2-pentenoyl-CoA + H2O ↔ (3 S)-3-hydroxyadipyl-CoA	4.2.1.17
				Adipyl-CoA + FAD ↔ 5-carboxy-2-pentenoyl-CoA + FADH_2_	1.3.99.-
				Adipate + CoA + ATP ↔ Adipyl-CoA + AMP + diphosphate	6.2.1.-
				Adipate semialdehyde + NADP+ + H_2_O ↔ adipate + NADPH + H^+^	1.2.1.4

Another mechanism is associated with NADH oxidization. Under micro-aerobic conditions, the cellular growth of microorganisms can be limited by NAD regeneration, which is necessary for glycolysis activity, and which occurs through NADH oxidization. Thus, when the heterologous reactions producing the targets are associated with NADH oxidization, these heterologous reactions are activated when the biomass production is maximized This phenomenon occurs, for example, in the production of (R)-propane-1,2-diol and 2-propyn-1-al.

We also found that some metabolites are produced only by *E. coli* under conditions of maximum biomass production, such as (R)-propane-1,2-diol and adipate semialdehyde. Unlike *S. cerevisiae* and *C. glutamicum*, *E. coli* possesses NAD transhydrogenase, which can convert NADP and NADH to NADPH and NAD  respectively (and vice versa). In *E. coli* cells, the excess NADH is converted to NADPH which can then enter the target production pathway.

### Differences in target production capacity among host microorganisms

**Figure 4:**
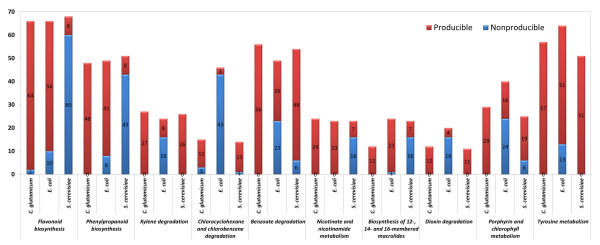
**The number of producible and non-producible metabolites in functional categories that exhibit significant differences between host microorganisms.** The blue and red bars represent the non-produced and produced metabolites respectively, under conditions of maximized biomass production.

While screening for heterologous pathways to produce the target metabolites discussed earlier, differences in production capacity between the three host microorganisms emerged; for example, a group of metabolites was inducible by the addition of heterologous reactions to one of the hosts, but was not produced by the other hosts. To characterize the differences in target production capacity, we categorized the producible metabolites (shown in the Additional files 5,6,7) using the KEGG Orthology database [16]. We then performed a chi-square statistical analysis to identify the categories in which the frequency of producible metabolites is significantly higher than expected. Figure 4 shows the 10 categories that demonstrated significant differences (*P* < 0.001). As shown in the figure, metabolites belonging to 5 categories, namely, “tyrosine metabolism,” “dioxin degradation,” “benzoate degradation,” “chlorocyclohexane and chlorobenzene degradation,” and “xylene degradation,” tended to be producible by *S. cerevisiae* and *C. glutamicum* but were scarce in *E. coli* cells.

Similarly, the metabolites in “flavonoid biosynthesis,” “phenylpropanoid biosynthesis,” and “nicotinate and nicotinamide metabolism” were preferentially generated by *E. coli* and *C. glutamicum*. Metabolites assigned to “porphyrin and chlorophyll metabolism” also tended to be produced in *C. glutamicum* cells. Likewise, the metabolites assigned to “biosynthesis of 12-, 14-, and 16-membered macrolides” were produced preferentially in *E. coli* cells. Such differences in production capabilities result from the different metabolic pathways by which the hosts produce necessary substrates, and from cellular compartmentalization in the yeast strain (which is absent in the bacterial strains).

In yeast cells, the compartments present barriers to metabolite transport. For instance, mitochondrial/cytoplasmic interfaces prohibit the production of certain target metabolites when sugar is used as a carbon source. Similarly, the production of metabolites in the “flavonoid biosynthesis” category was inhibited in yeast cells because the transportation of 4-coumarate between the mitochondria and the cytosol is not permitted; therefore, the yeast strain could not produce *p*-coumaroyl-CoA (required for making chalconoid, an important ingredient in flavonoid biosynthesis). Our genome-scale metabolic model does not account for transportation capabilities between compartments, which are currently unclear for many metabolites, and which might influence the production capacities of target metabolites in real cell systems.

## Conclusions

In conclusion, we developed a computational platform to investigate the extent to which industrial hosts can synthesize nonnative metabolites. Biosynthetic capabilities are evaluated by pathway design and flux calculations. We tested our platform using the industrial hosts *S. cerevisiae*, *E. coli*, and *C. glutamicum* as templates. Our results are consistent with those of earlier reports and provide additional alternative heterologous pathways. Producible nonnative metabolites predicted by our platform include industrial chemical compounds such as isoprene, α-farnesene, PHB, cadaverine, 1,3-propanediol, 1,2-propanediol, and vanillin. We propose that our platform is applicable to any genome-scale models that simulate cell factories. The platform greatly reduces the time and cost of heterologous pathway searching for target metabolites. Furthermore, appropriate expansions of the proposed system (for example, incorporating reaction irreversibility and source availability of heterologous enzymes), could significantly improve the scope of our system. We believe that this platform will accelerate the rational design of metabolic systems and thereby enhance microbial production of essential metabolites.

## Availability and requirements

The program for our pathway search algorithm is available at

http://www-shimizu.ist.osaka-u.ac.jp/pathway_search.zip. The program is written in Python. After extracting “pathway_search.zip”, the tool can be started by double clicking “runningScript.py” or by opening “runningScript.py” in Python IDLE, followed by pressing F5. All connectable nonnative metabolites including heterologous reaction are contained in the iteration folder. The folder input contains the necessary input files for identifying heterologous reactions of nonnative metabolites induced in a specified host.

## Competing interests

No competing interests declared.

## Authors’ contributions

SC constructed the algorithm and performed the simulations. CF participated in the design of the study and drafted the manuscript. HS conceived and supervised the study. All authors revised and approved the final manuscript.

## Supplementary Material

Additional file 1 **List of reactions used in this study.** The sheet “kegg_reaction_information” contains the metabolic reactions from the KEGG ligand database. Click here for file

Additional file 2 **List of connectable nonnative metabolites when *Corynebacterium glutamicum* was used as the host.** The sheet “C.glutamicum_connectable” contains all of the connected metabolites, including heterologous reaction(s), information about gene(s) from the KEGG database and the minimum *K*_m_ value from the BRENDA database. Click here for file

Additional file 3 **List of connectable nonnative metabolites when *Escherichia coli* was used as the host.** The sheet “E.coli_connectable” contains all of the connected metabolites, including heterologous reaction(s), information about gene(s) from the KEGG database and the minimum *K*_m_ value from the BRENDA database. Click here for file

Additional file 4 **List of connectable nonnative metabolites when *Saccharomyces cerevisiae* was used as the host.** The sheet “S.cerevisiae_connectable” contains all of the connected metabolites, including heterologous reaction(s), information about gene(s) from the KEGG database and the minimum *K*_m_ value from the BRENDA database. Click here for file

Additional file 5 **List of producible nonnative metabolites when*Corynebacterium glutamicum*was used as the host.**The sheet “C.glutamicum_maxTarget” contains all of the producible metabolites under the target maximization condition, including heterologous reaction(s), information about gene(s) from the KEGG database and the minimum *K*_m_ value from the BRENDA database. The sheet “C.glutamicum_maxBiomass” contains the producible metabolites under the biomass maximization condition, including heterologous reaction(s), information about gene(s) from the KEGG database and the minimum *K*_m_ value from the BRENDA database. Click here for file

Additional file 6 **List of producible nonnative metabolites when*Escherichia coli*was used as the host.**The sheet “E.coli_maxTarget” contains all of the producible metabolites under the target maximization condition, including heterologous reaction(s), information about gene(s) from the KEGG database and the minimum *K*_m_ value from the BRENDA database (nonstandard format). The sheet “E.coli_maxBiomass” contains the producible metabolites under the biomass maximization condition, including heterologous reaction(s), information about gene(s) from the KEGG database and the minimum *K*_m_ value from the BRENDA database. Click here for file

Additional file 7 **List of producible nonnative metabolite when *Saccharomyces cerevisiae* was used as the host.** The sheet “S.cerevisiae_maxTarget” contains all of the producible metabolites under the target maximization condition, including heterologous reaction(s), information about gene(s) from the KEGG database and the minimum *K*_m_ value from the BRENDA database. The sheet “S.cerevisiae_maxBiomass” contains the producible metabolites under the biomass maximization condition, including heterologous reaction(s), information about gene(s) from the KEGG database and the minimum *K*_m_ value from the BRENDA database. Click here for file
